# Coronary slow flow research: a bibliometric analysis

**DOI:** 10.1186/s40001-023-01326-w

**Published:** 2023-10-04

**Authors:** Jing Tong, Gui-Guang Bei, Li-Bo Zhang, Ben-Qiang Yang

**Affiliations:** Department of Radiology, General Hospital of Northern Theater Command, 83 Wenhua Road, Shenyang, 110016 Liaoning China

**Keywords:** Coronary slow flow, Bibliometrics, Research hotspots

## Abstract

**Background:**

Studies on coronary slow flow are receiving increasing attention, but objective evaluations are still lacking. The purpose of this study was to visualize the current status and research hotspots of coronary slow flow through bibliometric analysis.

**Methods:**

All relevant publications on coronary slow flow from 2003 to 2022 were extracted from the Web of Science Core Collection database and analyzed by VOSviewer and CiteSpace visualization software. Year of publication, journal, country/region, institution, and first author of each paper, as well as research hotspots were identified.

**Results:**

A total of 913 publications were retrieved. The journal with the most publications was Coronary Artery Disease. The country/region with the most publications was Turkey, followed by China and the United States. The institution with the largest publication volume was Turkey Specialized Higher Education Research Hospital. The author with the largest publication volume was Chun-Yan Ma from China. Keyword analysis indicated that “treatment and prognosis”, “pathogenesis and risk factors” and “diagnosis” were the clustering centers of coronary slow flow, and the research hotspots gradually changed with time, from pathogenesis to treatment and prognosis.

**Conclusion:**

Future research will focus on the search for effective and non-invasive detection indicators and treatments of coronary slow flow. Collaboration needs to be enhanced between different institutions or countries/regions, which would improve clinical outcomes for patients with coronary slow flow.

## Background

Coronary slow flow (CSF) was first proposed by Tambe et al. [1] in 1972, and its diagnosis was based on the results of coronary angiography. CSF is characterized by normal or near-normal epicardial coronary arteries (stenosis < 40%) with delayed distal vessel contrast opacification, as evidenced by either thrombolysis in myocardial infarction (TIMI) 2 flow or a corrected TIMI frame count > 27 frames (30 frames/s) in at least one epicardial vessel [2]. CSF can easily go unnoticed for a long time. Angina pectoris is the most common clinical presentation of CSF patients, which affects their quality of life and even leads to ventricular tachyarrhythmias or cardiac death [3–5]. Etiology and pathogenesis of CSF are not clearly defined, and there is no effective treatment. Many publications on CSF have been reported, but objective evaluation of the publications and comprehensive summary of research hotspots are still lacking.

Bibliometric analysis is a literature-mining method based on mathematics and statistics that can predict the research status and hotspots within specific domains through information visualization [6, 7]. Based on the Web of Science Core Collection (WoSCC) database, this paper attempts to explore the characteristics and development of publications on CSF from 2003 to 2022 with VOSviewer and CiteSpace visualization software to lay a foundation for future studies.

## Methods

### Data collection

We searched the WoSCC database from January 2003 to December 2022. The search strategy used to retrieve the data was TS = (coronary slow flow OR slow coronary flow). Only English articles of categories “Article” and “Review” were considered in this study.

### Data analysis

Bibliometric analysis was used to analyze the year of publication, journal, country/region, institution, and first author of each paper, as well as research hotspots of publications on CSF. The full records and cited references were exported from the WoSCC database in plain text format. VOSviewer software (version 1.6.18) was used to perform keyword co-occurrence analysis and generate visual maps. CiteSpace software (version 6.2. R2) was used to perform keyword burst analysis and explore the changing trend of publications on CSF.

## Results

### Annual distribution

**Fig. 1:**
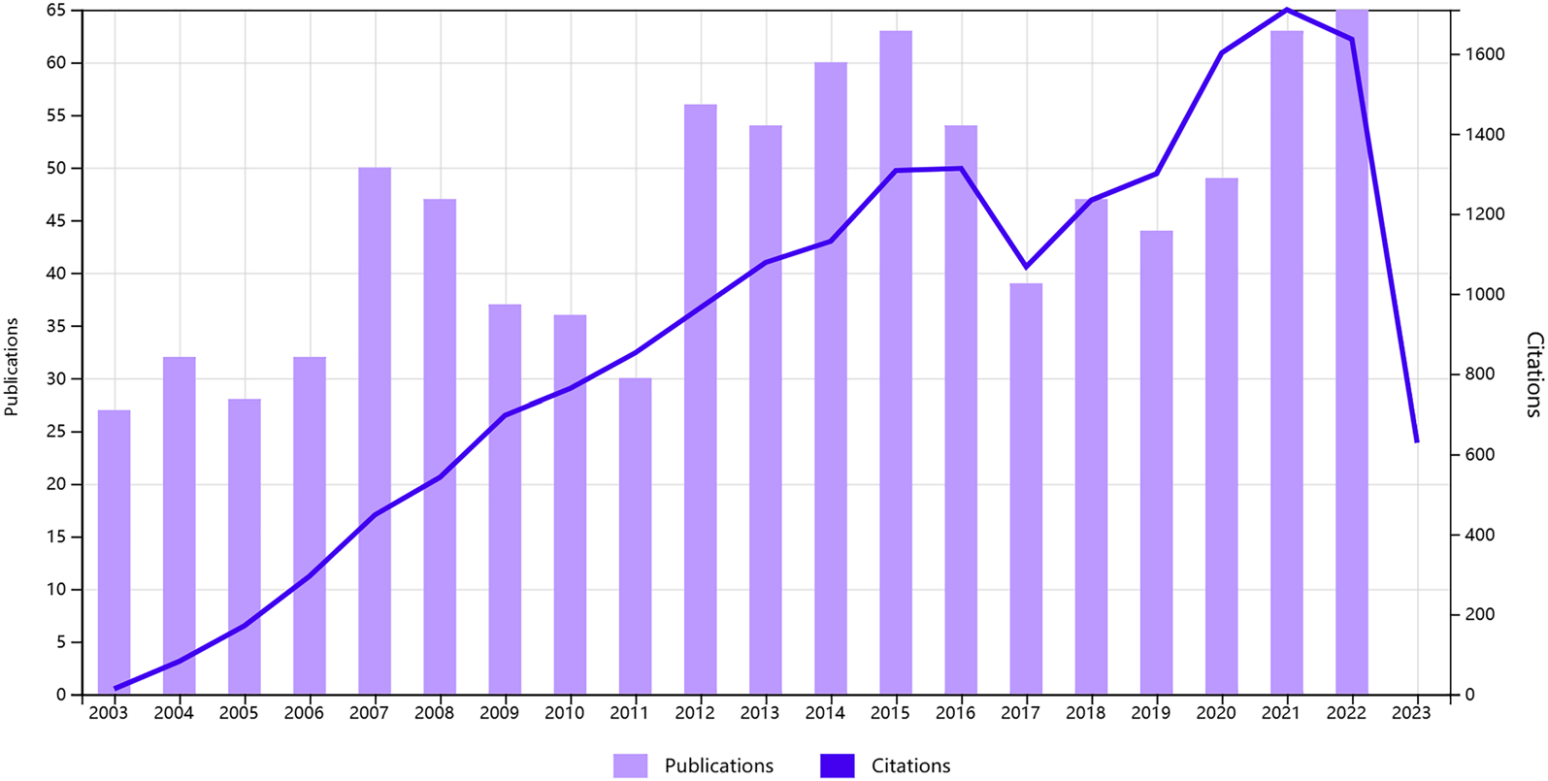
Annual growth trends of publications and total citations on CSF

A total of 913 publications on CSF were retrieved from the WoSCC database, including 835 primary articles and 78 reviews (Table 1). The publication volume exhibited a fluctuating increase from 2003 to 2015, decreased in 2016 and 2017, and increased rapidly from 2018 to 2022. The total citations increased year by year from 2003 to 2016, decreased in 2017, and increased rapidly from 2018 to 2022 (Fig. 1).

**Table 1:** Distribution of publications on CSF by year 2003–2022

Year	Publications (*n*), % of 913	Categories		Total citations
		Review	Article	
2003	27, 2.96%	1	26	13
2004	32, 3.50%	1	31	82
2005	28, 3.07%	0	28	171
2006	32, 3.50%	1	31	295
2007	50, 5.48%	4	46	448
2008	47, 5.15%	2	45	542
2009	37, 4.05%	3	34	697
2010	36, 3.94%	5	31	763
2011	30, 3.29%	4	26	852
2012	56, 6.13	5	51	965
2013	54, 5.91%	4	50	1078
2014	60, 6.57%	3	57	1131
2015	63, 6.90%	5	58	1308
2016	54, 5.91%	5	49	1313
2017	39, 4.27%	4	35	1067
2018	47, 5.15%	6	41	1234
2019	44, 4.82%	4	40	1300
2020	49, 5.37%	5	44	1602
2021	63, 6.90%	7	56	1710
2022	65, 7.12%	9	56	1636
Total	913, 100%	78	835	18,207

### Distribution of journals

The retrieved publications on CSF were published in 340 different journals, the top 15 of which by publication volume are listed in Table 2. The journals with the largest publication volume were Coronary Artery Disease, from the United States (*n* = 27), and the International Journal of Cardiology, from Ireland (*n* = 26). The journal with the highest impact factor (IF) was the Journal of the American College of Cardiology (IF = 24, 2022), which had the most total citations (1210).

**Table 2:** The top 15 journals by publication volume on CSF

Journal	Publications (*n*), % of 913	IF(2022)	Total citations	Country
Coronary Artery Disease	27, 2.96%	1.8	523	The USA
International Journal of Cardiology	26, 2.85%	3.5	567	Ireland
Catheterization and Cardiovascular Interventions	24, 2.63%	2.3	362	The USA
Heart and Vessels	19, 2.08%	1.5	303	Japan
Angiology	19, 2.08%	2.8	250	The USA
American Journal of Cardiology	16, 1.75%	2.8	289	The USA
BMC Cardiovascular Disorders	16, 1.75%	2.1	85	England
Journal of Invasive Cardiology	16, 1.75%	1.5	84	The USA
Clinical Hemorheology and Microcirculation	14, 1.53%	2.1	214	The Netherlands
Journal of the American College of Cardiology	13, 1.42%	24	1210	The USA
Circulation Journal	13, 1.42%	3.3	377	Japan
Kardiologia Polska	13, 1.42%	3.3	145	Poland
International Journal of Cardiovascular Imaging	13, 1.42%	2.1	100	The USA
International Heart Journal	12, 1.31%	1.5	198	Japan
Anatolian Journal of Cardiology	12, 1.31%	1.3	130	Turkey

*IF* impact factor

### Distribution of countries/regions

The retrieved publications on CSF were distributed among 56 countries/regions, the top 15 of which by publication number are listed in Table 3. The country/region with the largest publication volume was Turkey (*n* = 219), followed by China (*n* = 179) and the United States (*n* = 167).

**Table 3:** The top 15 countries/regions by publication volume on CSF

Countries/regions	Publications (*n*), % of 913	Total citations
Turkey	219, 23.99%	3720
Peoples Republic of China	179, 19.61%	1833
The USA	167, 18.29%	5969
Japan	86, 9.42%	2042
Italy	64, 7.01%	2180
Germany	43, 4.71%	1288
England	34, 3.72%	1535
Canada	23, 2.52%	864
Australia	23, 2.52%	785
France	22, 2.41%	778
South Korea	19, 2.08%	291
The Netherlands	17, 1.86%	527
Iran	17, 1.86%	133
Poland	16, 1.75%	228
Israel	13, 1.42%	229

**Fig. 2:**
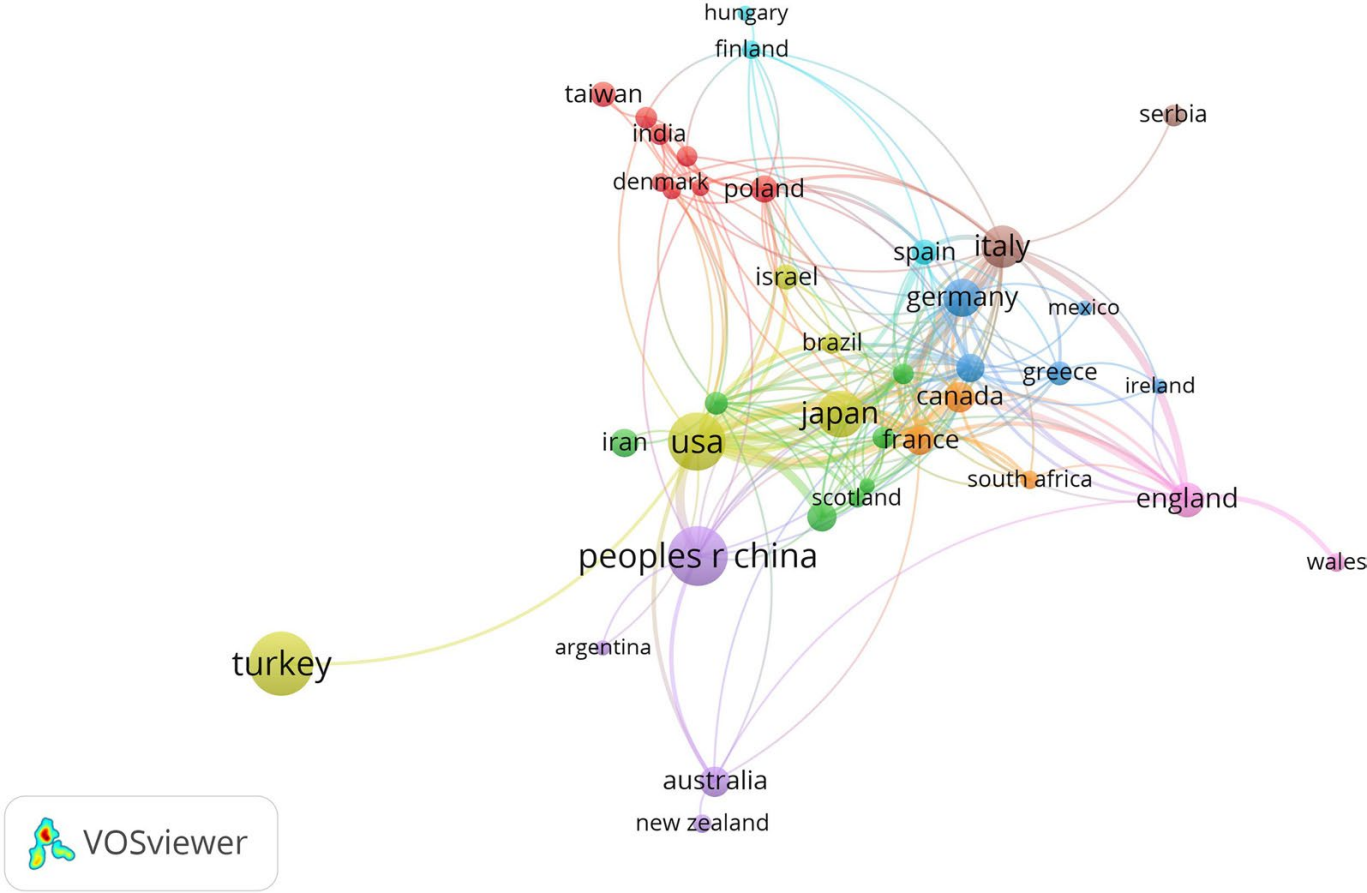
The co-operation network of countries/regions on CSF publications

VOSviewer software was used to generate a visual map of countries/regions. Figure 2 shows the co-operation networks and relationships between different countries/regions, presented by different-colored nodes and lines. Identically colored nodes indicate countries/regions with a higher frequency of co-occurrence and closer co-operative relationships, putting them in the same cluster. The node size is proportional to the publication volume. The thickness of the line indicates the strength of the co-operative relationship. Forty countries/regions had 3 or more publications, of which the USA had the highest total link strength (total link strength = 113), followed by Italy (total link strength = 55).

### Distribution of institutions

The retrieved publications on CSF were distributed among 1379 institutions, the top 15 of which by publication volume are listed in Table 4. The institution with the largest publication volume was Turkey Specialized Higher Education Research Hospital (*n* = 22), followed by Capital Medical University (*n* = 21).

**Table 4:** The top 15 institutions by publication volume on CSF

Institution	Publications (*n*), % of 913	Total citations	Country
Turkey Specialized Higher Education Research Hospital	22, 2.41%	646	Turkey
Capital Medical University	21, 2.30%	182	China
China Medical University	17, 1.86%	123	China
Icahn School of Medicine at Mount Sinai	14, 1.53%	569	The USA
Inonu University	14, 1.53%	562	Turkey
Jichi Medical University	14, 1.53%	296	Japan
University of California Los Angeles	14, 1.53%	244	The USA
Harvard University	13, 1.42%	636	The USA
Baskent University	12, 1.31%	502	Turkey
Chinese Academy of Medical Sciences Peking Union Medical College	12, 1.31%	232	China
Pamukkale University	11, 1.20%	224	Turkey
Tel Aviv University	11, 1.20%	191	Israel
Dr. Siyami Ersek Cardiac Vascular Surgery Training Research Hospital	11, 1.20%	151	Turkey
Ondokuz Mayis University	11, 1.20%	84	Turkey
Mersin University	10, 1.10%	458	Turkey

**Fig. 3:**
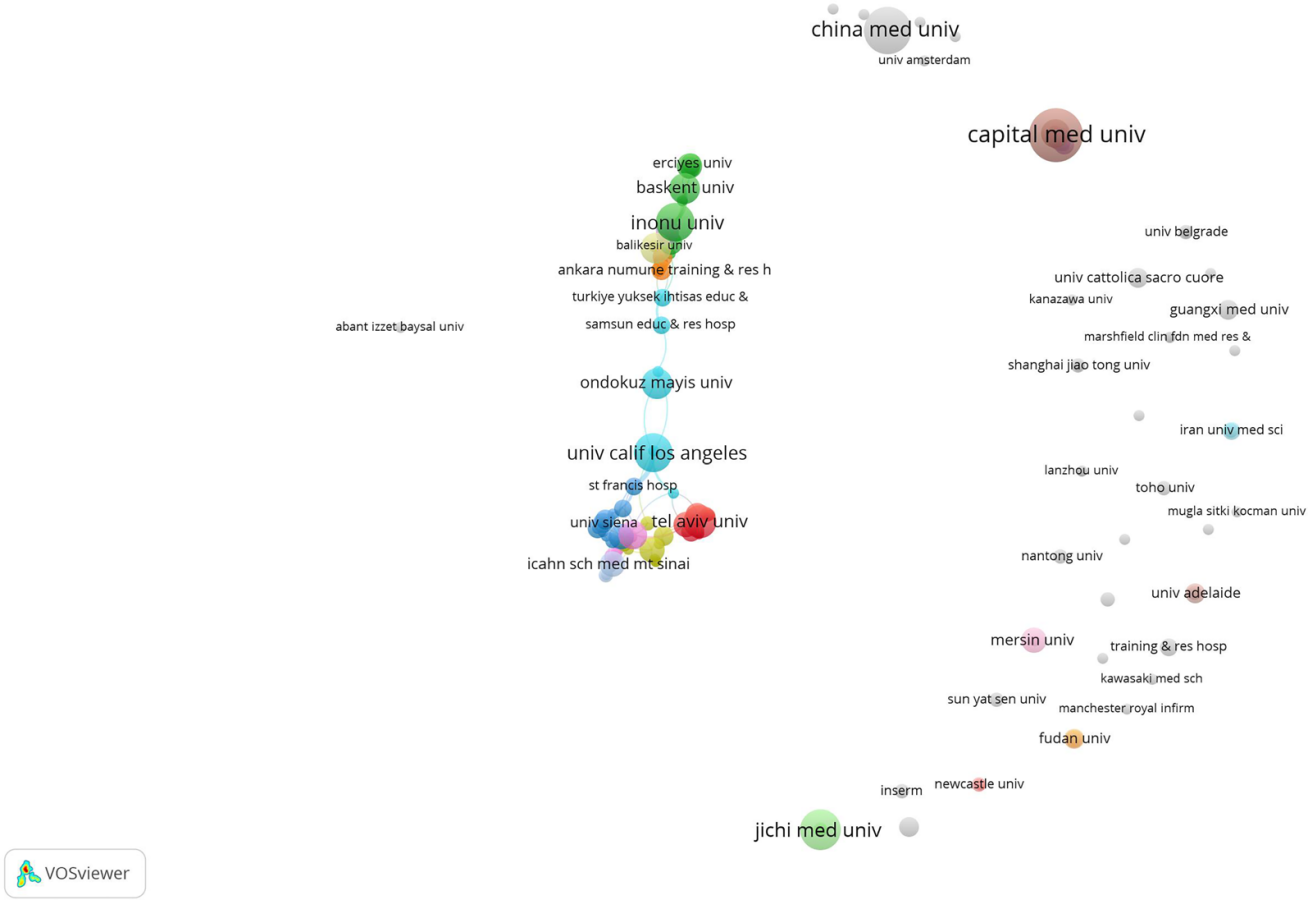
The co-operation network of institutions on CSF publications

VOSviewer software was used to generate a visual map of institutions. Figure 3 shows the co-operation networks and relationships between different institutions presented by different-colored nodes and lines. Identically colored nodes indicate institutions with close co-operative relationships belonging to the same cluster. The node size is proportional to the publication volume. The thicker the line, the stronger the co-operative relationship between different institutions. A total of 145 institutions had 3 or more publications, of which Columbia University had the highest total link strength (publication volume = 9, total link strength = 25), followed by the Cardiovascular Research Foundation (publication volume = 9, total link strength = 23).

### Distribution of authors

The retrieved publications on CSF included 4892 authors, the top 15 of whom by publication number are listed in Table 5. The author with the largest publication volume was Chun-Yan Ma from China (*n* = 14), followed by Mustafa Cetin from Turkey (*n* = 13) and Yong-Huai Wang from China (*n* = 12).

**Table 5:** The top 15 authors by publication volume on CSF

Author	Publications (*n*), % of 913	Total citations	Country
Chun-Yan Ma	14, 1.53%	112	China
Mustafa Cetin	13, 1.42%	207	Turkey
Yong-Huai Wang	12, 1.31%	100	China
Alpay Turan Sezgin	11, 1.21%	481	Turkey
Irfan Barutcu	11, 1.21%	384	Turkey
Kenichi Sakakura	11, 1.21%	172	Japan
Hiroshi Wada	11, 1.21%	161	Japan
Hideo Fujita	10, 1.10%	117	Japan
Dilek Ciçek	9, 0.99%	421	Turkey
Ahmet Camsari	9, 0.99%	351	Turkey
Hakan Gullu	9, 0.99%	305	Turkey
Tommaso Gori	9, 0.99%	211	Italy
Shin-ichi Momomura	9, 0.99%	160	Japan
Yousuke Taniguchi	9, 0.99%	101	Japan
Kei Yamamoto	9, 0.99%	101	Japan

**Fig. 4:**
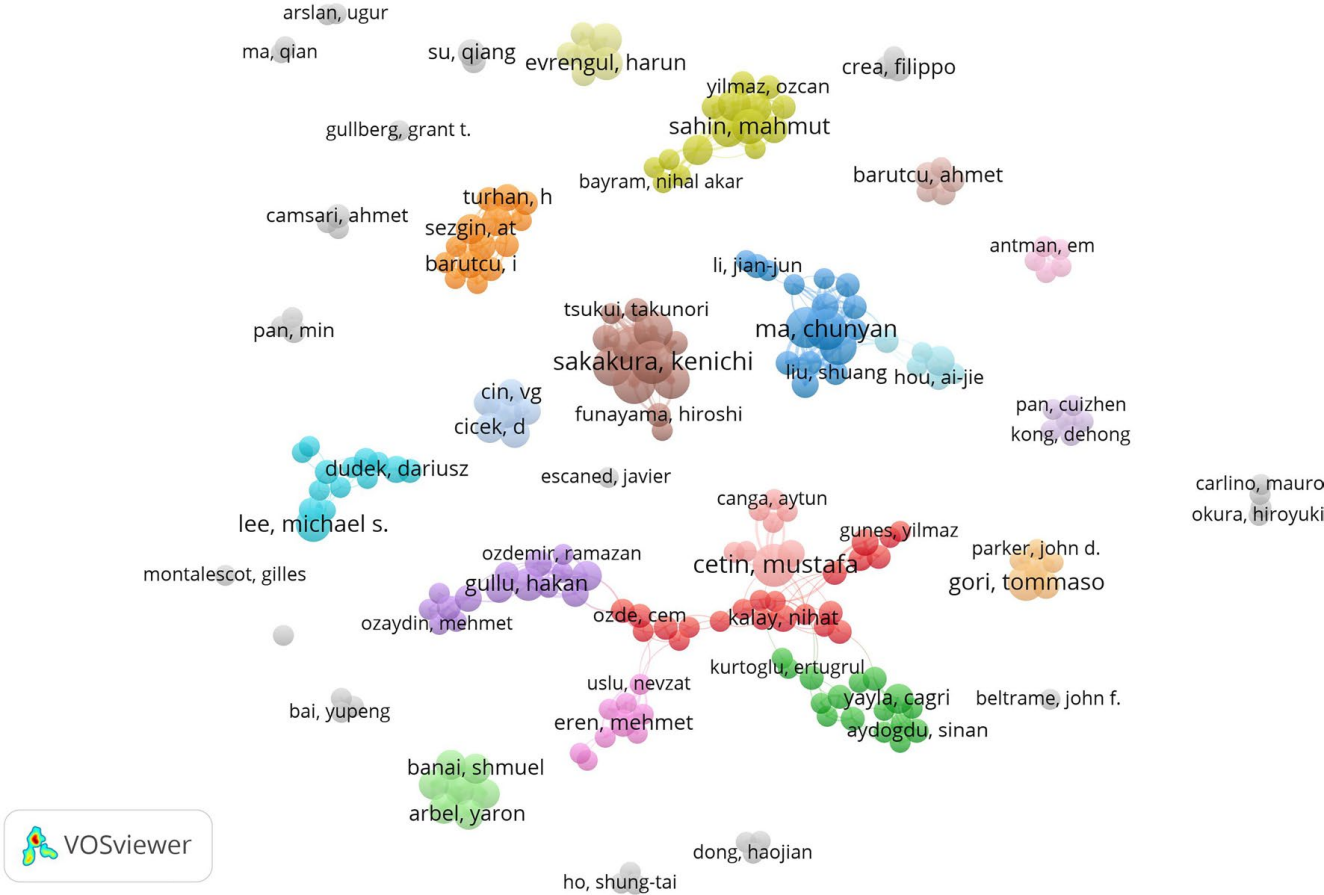
The co-operation network of authors on CSF publications

VOSviewer software was used to generate a visual map of the authors. Figure 4 shows the co-operation networks and relationships between different authors represented by different-colored nodes and lines. Identically colored nodes indicate authors with close co-operative relationships belonging to the same cluster. The node size is proportional to the publication volume. The thicker the line, the stronger the co-operative relationship between different authors. A total of 227 authors had 3 or more publications, of whom Kenichi Sakakura and Hiroshi Wada from Japan had the highest total link strength (total link strength = 70).

### Research hotspots and frontiers

#### Keyword co-occurrence analysis

We conducted keyword co-occurrence analysis to reveal the research hotspots on CSF via VOSviewer software. The small clusters of keywords that appeared fewer than 10 times were filtered out, and a keyword co-occurrence map of CSF high-frequency keywords containing 3 main clusters was generated.

**Fig. 5:**
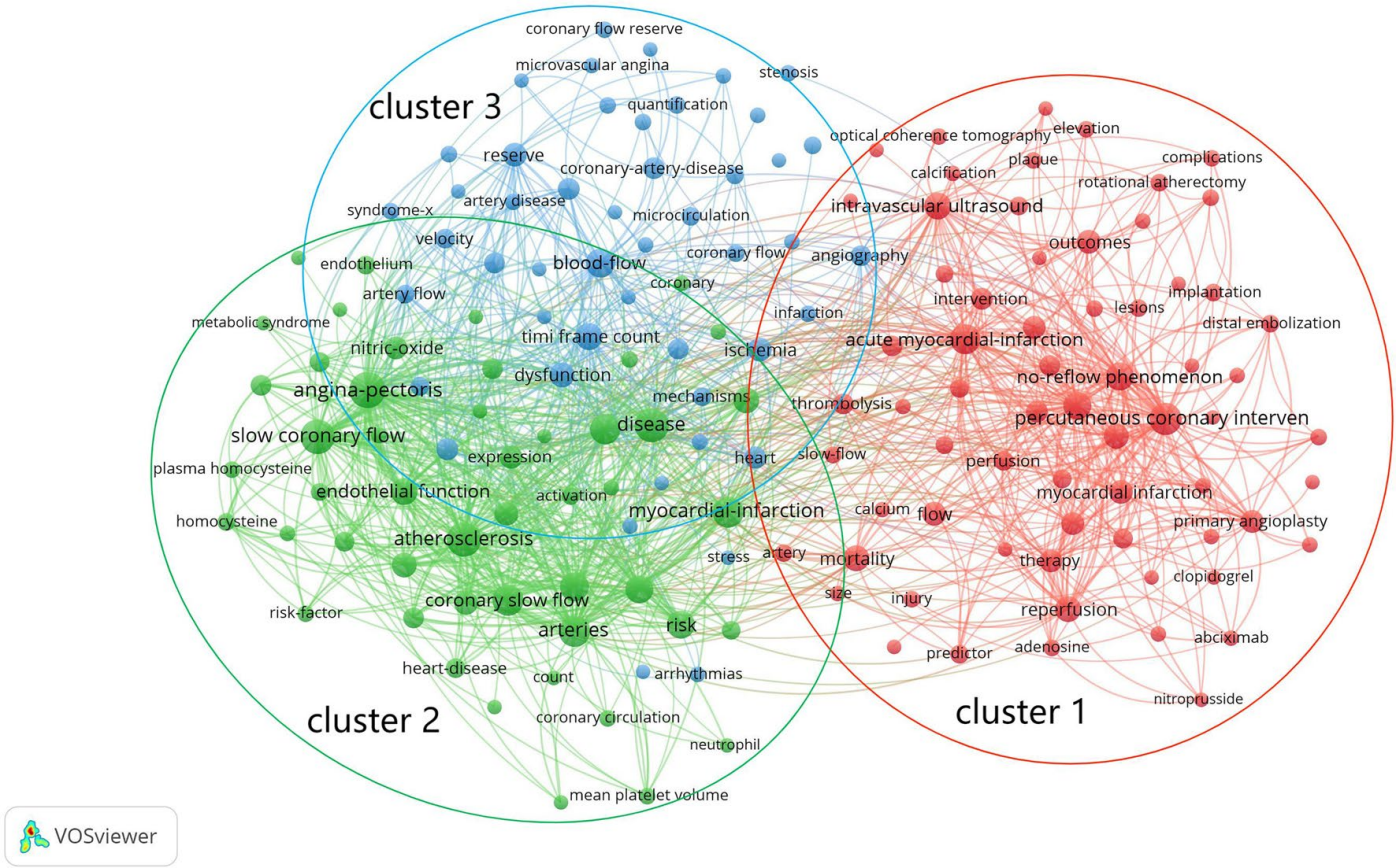
Keyword co-occurrence map on CSF

In Fig. 5, nodes represent keywords, and identically colored nodes belong to the same cluster. The keywords in the same cluster fall under similar research directions. Cluster 1 (red) is summarized as “treatment and prognosis”, which covers 67 keywords, including percutaneous coronary intervention (PCI), acute myocardial infarction, no-reflow phenomenon, angioplasty, intravascular ultrasound, mortality, reperfusion, outcomes, therapy, intervention, etc. Cluster 2 (green) is summarized as “pathogenesis and risk factors”, which covers 49 keywords, including slow coronary flow, atherosclerosis, artery disease, coronary slow flow, inflammation, risk, endothelial function, frame count, C-reactive protein, nitric oxide, etc. Cluster 3 (blue) is summarized as “diagnosis”, which covers 48 keywords, including blood flow, TIMI frame count, dysfunction, ischemia, coronary flow reserve, heart, coronary artery disease, angina, echocardiography, coronary angiography, etc.

#### Keyword burst analysis

**Fig. 6:**
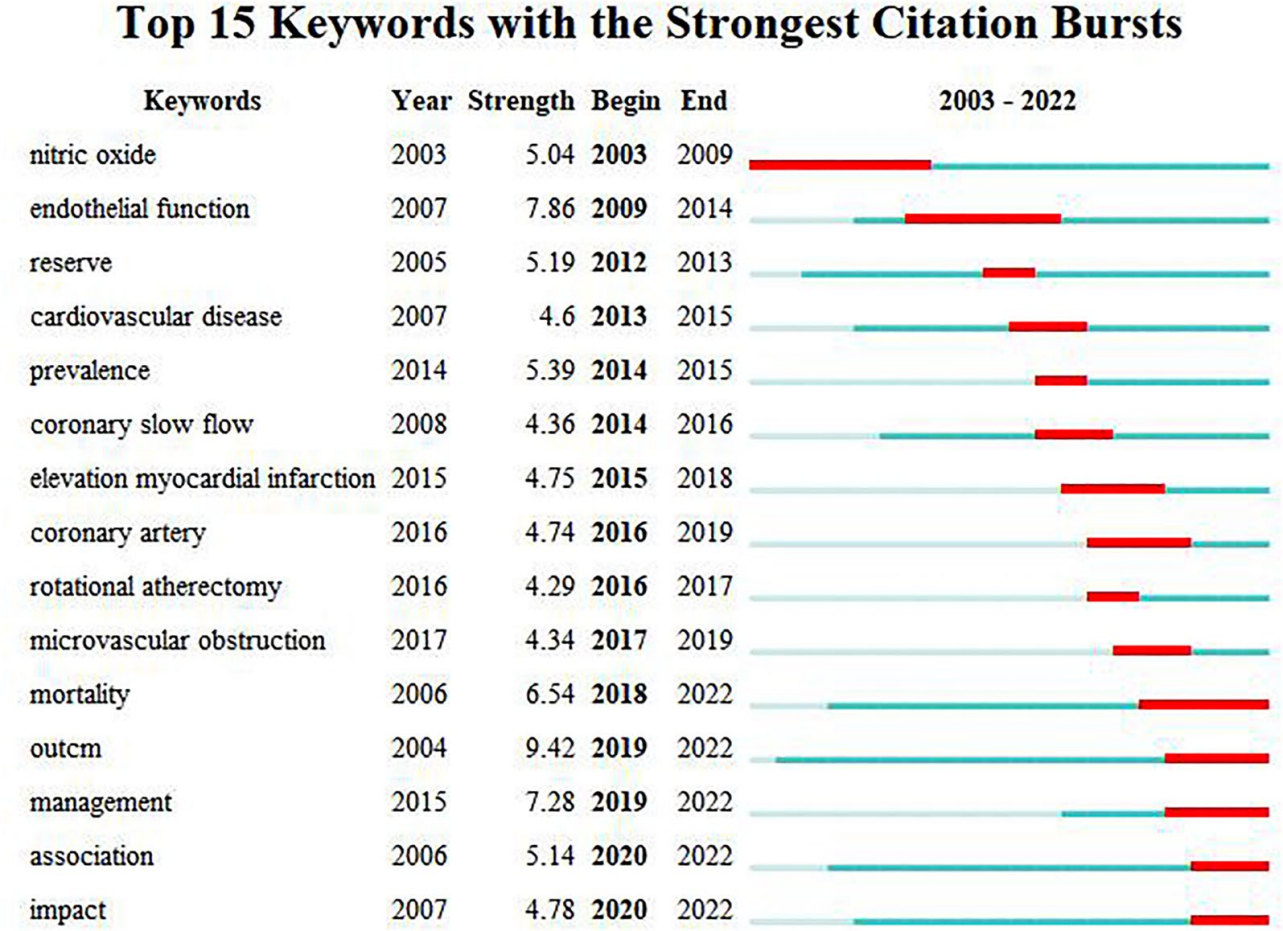
The 15 keywords with the strongest citation bursts had an annual change of strength

We conducted keyword burst analysis to reveal the changing trend of publications and research frontiers on CSF via CiteSpace software. The 15 keywords with the strongest citation bursts are shown in Fig. 6. The blue line represents the timeline from 2003 to 2022, while the red line represents the length of each burst. Before 2015, studies focused on the pathogenesis of CSF, which was primarily associated with endothelial function and coronary flow reserve. The keywords in this period included nitric oxide, endothelial function, reserve, etc. After 2015, studies focused on disease-related research. Among them, studies focused on ST-elevation myocardial infarction and rotational atherectomy from 2015 to 2017, while studies focused on the treatment and prognosis of CSF from 2018 to 2022. The keywords in this period included ST-elevation myocardial infarction, coronary artery, rotational atherectomy, microvascular obstruction, mortality, outcome, management, association, and impact.

## Discussion

It has been more than 50 years since the first detection of CSF [1]. As coronary arteries are not significantly stenotic during coronary angiography in patients with CSF, CSF has not attracted enough attention for a considerable time. However, CSF patients may develop recurrent symptoms of angina pectoris, and even life-threatening cardiovascular events can occur in severe cases, so studies on CSF are receiving increasing attention [5]. To the best of our knowledge, this study is the first bibliometric analysis of CSF. It reflects the current status and research hotspots of CSF in an objective and comprehensive way, which we hope will promote further studies in this field.

This study found that the author with the most publications was Chun-Yan Ma from China. The studies of her team have mainly concentrated on the novel echocardiography-based technique to assess CSF and gene polymorphisms in the pathogenesis of CSF [8, 9]. Six of the top 15 authors by publications on CSF were from Turkey, and some authors had very close co-operative relationships. Among them, Mustafa Cetin, Alpay Turan Sezgin, and Irfan Barutcu ranked in the top 5 authors by publication number. Their research has a wide scope, covering pathogenesis, risk factors, cardiac electrophysiology, ultrasound assessment, treatment, etc. [10–12]. We listed the top 15 countries/regions and institutions by publication volume, while the co-operative relationships between different countries/regions and institutions were visualized as visual maps. International co-operation between countries/regions, institutions, and authors should be further enhanced in the field of CSF. This study also compiled quantitative information about journals, which would be helpful in tracking research dynamics and selecting target journals.

The research direction of cluster 1 in the keyword co-occurrence analysis was associated with the treatment and prognosis of CSF. Our keyword burst analysis revealed that the treatment and prognosis of CSF was the main research frontier in recent years, with keywords, such as mortality, outcome, and management. Most studies have focused on pharmacotherapy of CSF, but there is still no safe and effective drug supported by evidence-based medicine. A growing number of studies have found that statin therapy can improve coronary blood flow and coronary flow reserve [5, 13]. Niu et al. [14] performed a prospective randomized trial of 108 patients with CSF and found that atorvastatin could treat CSF by improving endothelial function. Some studies found that dipyridamole and nicorandil could improve left ventricular systolic and diastolic function in patients with CSF [15, 16]. Several other treatments, such as traditional Chinese medicine, hyperbaric oxygen therapy, and cardiac rehabilitation, remain in the exploratory stage [17–19].

The high-frequency keywords in cluster 1 included PCI, acute myocardial infarction, and no-reflow phenomenon. Slow flow/no-reflow phenomenon after PCI in myocardial infarction was one main focus of research on CSF. Shah et al. [20] found that predictors built on the basis of history and angiographical features could predict the occurrence of slow flow/no-reflow phenomenon after primary PCI. Reddy et al. [21] found that higher necrotic core volume detected by intravascular ultrasound and virtual histology might be a potential risk factor for CSF phenomenon after PCI in patients with ST-elevation myocardial infarction. Carrick et al. [22] found that deferred stenting might reduce the slow flow/no-reflow phenomenon in primary PCI and increase myocardial salvage.

Cluster 2 in our keyword co-occurrence analysis was associated with the pathogenesis and risk factors for CSF, including keywords, such as atherosclerosis, inflammation and endothelial function. The keywords in the keyword burst analysis were nitric oxide and endothelial function from 2003 to 2014. Earlier studies on the pathogenesis of CSF focused on endothelial dysfunction [23]. A variety of factors can cause endothelial dysfunction, most importantly the imbalance between the production of vasodilatory factors, such as nitric oxide and vasoconstrictive factors such as endothelin [24]. Some studies found that reduced plasma nitric oxide and elevated plasma endothelin-1 were prevalent in patients with CSF [25, 26]. In recent years, an increasing number of studies have found a potential role for factors, such as atherosclerosis, inflammation, gene polymorphisms, and genetic predisposition in the pathogenesis of CSF [27, 28]. Tapar et al. [29] found that CSF could be considered a subgroup of coronary artery diseases with increased intimal thickness in coronary arteries and extensive calcification. Some studies found that inflammatory markers, such as C-reactive protein and the neutrophil-to-lymphocyte ratio, in patients with CSF were significantly elevated compared with patients with normal coronary flow [30, 31]. An in-depth study on the pathogenesis of CSF could provide better evidence for individualized prevention and treatment.

The diagnostic methods of CSF have not received enough attention in previous studies. The diagnosis of CSF is based on coronary angiography. Since coronary angiography is invasive and expensive, the diagnosis and follow-up of CSF are difficult. A series of explorations have been performed to find non-invasive diagnostic markers of CSF [32, 33]. These markers are relatively limited due to their inadequate specificity. Research on novel echocardiography-based techniques for the diagnosis and evaluation of CSF has become a research hotspot in recent years [34, 35], while the application of other imaging methods in the clinical and scientific research fields of CSF is rarely reported. Thus, non-invasive diagnosis of CSF is recommended for future in-depth studies.

There are some limitations to this study. First, only the publications in the WoSCC database were searched, which may have caused some biases in the results. Second, with the rapid updating of publications in the field of CSF, our literature search may have missed some research hotspots.

## Conclusions

The study of CSF is of great clinical value. This bibliometric analysis of the literature on CSF reflects the research status and hotspots over the last two decades, visualized with dedicated software. The Journal Coronary Artery Disease, country/region Turkey, and author Chun-Yan Ma were top ranked. Collaboration needs to be enhanced between different countries/regions and institutions, which would improve clinical outcomes for patients with CSF. “Treatment and prognosis”, “pathogenesis and risk factors”, and “diagnosis” were the clustering centers of CSF in the keyword co-occurrence analysis. The research focus gradually moved from pathogenesis to disease-related research to treatment and prognosis over the years. The search for effective and non-invasive detection indicators and treatments of CSF will be the future trends in research. This study will help researchers find relevant literature and academic partners, offer a direction for journal submissions, and provide a reference for the identification of hotspots in the field of CSF.

## Data Availability

The datasets used and/or analyzed during the current study are available from the corresponding author on reasonable request.

## Abbreviations

CSF: Coronary slow flow
TIMI: Thrombolysis in myocardial infarction
WoSCC: Web of Science Core Collection
IF: Impact factor
PCI: Percutaneous coronary intervention
